# Detrending Technique for Denoising in CW Radar

**DOI:** 10.3390/s21196376

**Published:** 2021-09-24

**Authors:** In-Seong Lee, Jae-Hyun Park, Jong-Ryul Yang

**Affiliations:** Department of Electronic Engineering, Yeungnam University, Gyeongsan 38541, Gyeongbuk, Korea; dldlstjd0322@yu.ac.kr (I.-S.L.); bravopark@ynu.ac.kr (J.-H.P.)

**Keywords:** circle fitting method, continuous-wave radar, DC drift, DC offset, denoising, detrending, displacement measurement, I/Q calibration, vital signal detection

## Abstract

A detrending technique is proposed for continuous-wave (CW) radar to remove the effects of direct current (DC) offset, including DC drift, which is a very slow noise that appears near DC. DC drift is mainly caused by unwanted vibrations (generated by the radar itself, target objects, or surroundings) or characteristic changes in components in the radar owing to internal heating. It reduces the accuracy of the circle fitting method required for I/Q imbalance calibration and DC offset removal. The proposed technique effectively removes DC drift from the time-domain waveform of the baseband signals obtained for a certain time using polynomial fitting. The accuracy improvement in the circle fitting by the proposed technique using a 5.8 GHz CW radar decreases the error in the displacement measurement and increases the signal-to-noise ratio (SNR) in vital signal detection. The measurement results using a 5.8 GHz radar show that the proposed technique using a fifth-order polynomial fitting decreased the displacement error from 1.34 mm to 0.62 mm on average when the target was at a distance of 1 m. For a subject at a distance of 0.8 m, the measured SNR improved by 7.2 dB for respiration and 6.6 dB for heartbeat.

## 1. Introduction

A continuous-wave (CW) radar, which can be implemented with a simple hardware configuration, can detect movement; speed; and vital signals, such as respiration and heartbeat, from the Doppler frequency [1,2,3,4,5,6,7,8,9]. The CW radar can be extended to a frequency-shift keying (FSK) radar based on a similar hardware configuration. This can measure an absolute distance in a short range by using the phase differences between the transmitted and received signals obtained by continuously switching between two or more CW signals with different frequencies [10,11,12].

The phase-detection accuracy of CW/FSK radars determines the detection performance of the displacement measurement with sub-wavelength precision and the vital signal detection obtained from the movement of the surface of the human body [13,14]. A circle fitting method has been proposed to reduce the error in detecting the phase difference caused by the non-ideal characteristics of clutter in the surroundings or radar hardware and the imbalance between in-phase (I) and quadrature (Q) signals [15,16,17]. This method can increase the accuracy of the phase measurement in the baseband by approximating the trajectory of the measured I/Q signals to a circle [18]. In this method, the direct current (DC) offset can be removed by moving the center of the estimated circle to the center of the complex plane, and the I/Q imbalance can be corrected by compensating the ellipse trajectory with a circle [19,20]. Previous studies on the implementation of the circle fitting method generally assume that the DC offset and I/Q imbalance are almost constant while drawing the trajectory of the circle in the baseband of the CW radar [21,22,23]. This assumption can be accepted in general for an I/Q imbalance caused by hardware imperfections but not for a DC offset generated by clutters in the surroundings and device characteristics in the radar [24,25,26]. In particular, changes in the characteristics of components due to heat generation, vibrations at a very low frequency, and unintentional movements of a subject with limited movement can generate the so-called DC drift phenomenon, which is the slow movement of the DC offset [27,28]. DC drift lowers the phase-detection accuracy in CW/FSK radars because it makes it difficult for the DC offset to be removed in the circle fitting method [29]. The signal-to-noise ratio (SNR) of the vital signal detection, the accuracy of the displacement measurement in the CW radar, and the accuracy of the range finding in the FSK radar decrease due to the lowered accuracy in phase detection.

In this study, a detrending technique that can compensate for the effect of DC drift is proposed to improve the phase-detection accuracy of the circle fitting method in CW radars. The proposed technique models and removes DC drift in the baseband signals obtained for a certain period of time by polynomial fitting. The simulation results for respiration detection using CW radar indicate that the proposed technique can remove the effect of DC drift, which is present in signals with a frequency 10 times lower than the desired signal. The measurement results of the displacement measurement of 0.3 m for a target object located at 1 m and the respiration and heartbeat detection for the subject at 0.8 m show that the proposed technique can eliminate DC drift in CW radars. The effect of DC drift in CW/FSK radars and the principle of the proposed detrending technique are described in Section 2. The simulation results in Section 3 show that the proposed detrending technique effectively cancels the DC drift modeled to periodic signals with a very low frequency. Section 4 presents the experimental setup for the validation of the proposed technique with displacement measurement and vital signal detection in the CW radar. The effects of the proposed technique are discussed using the CW radar measurement results in Section 5. The conclusions are presented in Section 6.

## 2. Detrending Technique

### 2.1. DC Offset and Drift in the CW Radar

When the residual phase noise in each channel is neglected by the radar correlation effect, the baseband I/Q signals *I*(*t*) and *Q*(*t*) in the CW radar can be expressed as

(1) $$I\left(t\right)=A_{I}\cos\left(\frac{4\pi x\left(t\right)}{\lambda }+\frac{4\pi d_{0}}{\lambda }\right)+DC_{I}\left(t\right),$$

(2) $$Q\left(t\right)=A_{Q}sin\left(\frac{4\pi x\left(t\right)}{\lambda }+\frac{4\pi d_{0}}{\lambda }+\varphi_{E}\right)+DC_{Q}\left(t\right),$$

where λ is the wavelength of the operating frequency, $A_{I}\;$ and $A_{Q}$ represent the amplitude of I/Q signals, *d_0_* is the distance from the radar to the target, φ_E_ is the phase offset between the I and Q channels, and *x*(*t*) is the displacement of the target object. The measured data from the CW radar are *x*(*t*), and they are obtained from the phase change of the trigonometric function of I/Q signals, which vary with time. The FSK radar can additionally detect *d*_0_ from the change in phase difference obtained using two frequencies [10]. The DC offset in each channel, *DC_I_*(*t*) and *DC_Q_*(*t*), can be expressed with two terms depending on the changes with time as follows:

(3) $$DC_{I}\left(t\right)=i_{DC}\left(t\right)+I_{DC},$$

(4) $$DC_{Q}\left(t\right)=q_{DC}\left(t\right)+Q_{DC},$$

where *i_DC_*(*t*) and *q_DC_*(*t*) are the time-variant DC offset voltages, and *I_DC_* and *Q_DC_* are the static DC offset voltages in the I/Q baseband signals. *I_DC_* and *Q_DC_*, or some *i_DC_*(*t*) and *q_DC_*(*t*), which can be averaged for a certain period of time, can be easily removed using the calibration process in the digital domain, which moves the center of a circle or arc in the complex plane [30]. In addition, if the desired signal can be distinguished from the frequency of the time-variant DC offset due to the difference in the frequency domain, the DC offset can be easily removed by a high-pass filter in the baseband. However, DC offset removal by a high-pass filter can attenuate the level of the desired signal located at a low frequency, such as respiration and heartbeat signals. When the DC offset has periodicity at a very low frequency or does not converge for a certain time, the center of a circle or arc in the process appears to move, and the accuracy of extracting the phase variation decreases.

**Figure 1:**
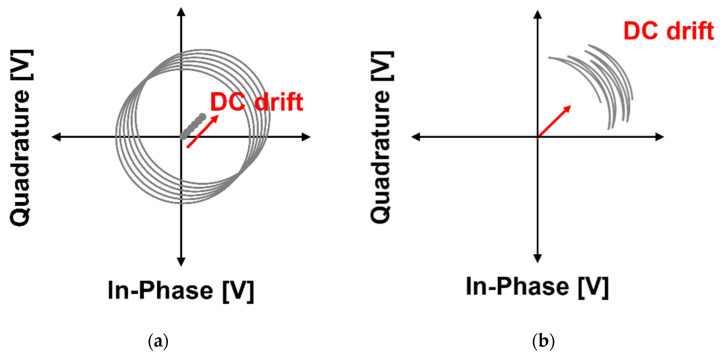
DC drift of the baseband I/Q signals in the complex plane: (**a**) when the displacement *x*(*t*) is sufficiently longer than the wavelength of the transmitting signals in the radar; (**b**) when *x*(*t*) is shorter than the wavelength.

DC drift is defined as a phenomenon in which the DC offset moves in a certain direction in the complex plane due to a very slow change. DC drift is presented as two cases, as shown in Figure 1, depending on the magnitude of *x(t)* compared to the wavelength of the operating frequency. The two cases might be understood as the same phenomenon in which the centers of the circles and arcs move in one direction at a very slow speed, but the algorithm to remove the effect of the DC drift cannot be the same between the two cases. The DC drift in Figure 1a is only presented by the center movement of the circle due to the long *x(t)* compared to the wavelength, and the DC drift can be compensated using the results of the method that estimated the vector for the movement of the center of a circle. However, when the method is used to remove the DC drift, the accuracy of the displacement or phase measurement deteriorates due to the time variation of the arc length generated by the DC drift as shown in Figure 1b. The variation in the arc length due to DC drift also occurs in the case of Figure 1a, but it is not necessary to obtain the variation in length because the accuracy of the circle fitting method is high due to the drawing of a perfectly circular trajectory. In this case, it is difficult to estimate the variation in the arc length in general. The phenomenon shown in Figure 1b is generated not only by the short length difference between the wavelength of the operating frequency and the desired displacement of the target but also by the characteristic change in the radar components due to heat generation and the ambient vibration in the very low frequency band [27,29]. In particular, the detection accuracy of the vital sign detection using the CW/FSK radar can be lowered by the DC drift generated by undesired movement of the subject in the radar sensor. Therefore, a generalized compensation technique for DC drift removal is required to improve the measurement accuracy of CW/FSK radars caused by changes at very slow frequencies or non-converged characteristics regardless of the displacement magnitude of the target object.

### 2.2. Detrending Technique Based on Polynomial Fitting in Time Domain 

**Figure 2:**
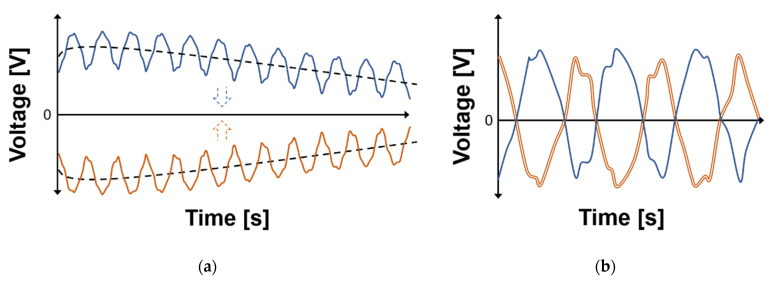
Baseband I/Q signals in time domain: (**a**) Raw waveform in each I/Q channel, including DC drift. The dash lines show the trends by DC drift; (**b**) calibrated waveform in each I/Q channel.

The detrending technique is defined as removing the effect of DC drift in the I/Q baseband signals. As shown in Figure 2, the DC drift with a very low frequency component appears to change more slowly than the I/Q baseband signals in the time domain. DC drift in the form of non-convergence also appears similar to the case of the DC drift with a very low frequency component when the baseband signals, including DC drift, are not saturated at the input of the analog-to-digital converter (ADC). Figure 2a shows that different DC offsets and drifts occur in each channel due to the imperfections of the radar hardware module and the effects of the parasitic components in the module. As shown in Figure 2b, the proposed detrending technique effectively removes the DC or near DC components in the I and Q channels by obtaining the model equation using polynomial fitting and calibrating the original signals with the equation. The calibrated I/Q signals without DC or near DC components can greatly improve the accuracy of phase extraction through simple digital signal processing with a circle fitting algorithm.

**Figure 3:**
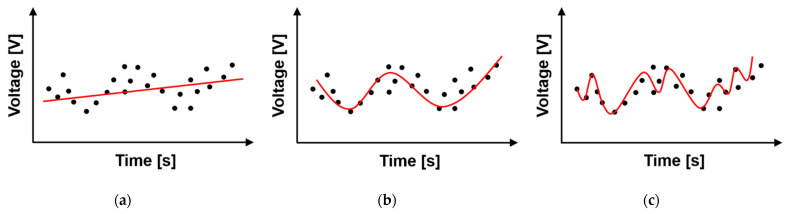
Trend lines of the DC drift in the proposed detrending technique depending on the polynomial order: (**a**) under fitting; (**b**) best fitting; (**c**) over fitting.

The generated DC drift in each channel of a single radar module is not exactly the same because DC drift appears individually, but there is a correlation between the DC drift of I/Q signals because the effect on DC drift is within the radar module. This correlation indicates that the DC drift in each channel can be modeled by polynomial fitting with the same order for a specific time period. The polynomial fitting for the input *x* can be expressed by the fitting equation *f(x)* as follows:

(5) $$f\left(x\right)=\overset{n}{\underset{i=0}{\sum }}\beta_{i}\cdot x^{i}\;$$

where *n* is the maximum order of the polynomial fitting, and *β_i_* is the coefficient of the *ith* order polynomial. The time period and the maximum order of the polynomial are the most important factors for estimating the DC drift in the baseband signals. These parameters are closely related to each other in the implementation of the signal process because the signal components to be removed by the proposed detrending technique are mainly located in the frequency band below 0.1 Hz. When the polynomial fitting is performed with a low order to the sampled baseband signals in the proposed technique, the DC drift cannot be effectively extracted, as shown in Figure 3a. When a high-order polynomial is used to fit the DC drift in the proposed technique, as shown in Figure 3c, the desired signals, especially at the initial and final data, can be lost by the signal process. Therefore, it is important for the proposed detrending technique to determine the best fitting order of the polynomial that can remove only the DC drift, as shown in Figure 3b. Considering the measurement environment, target object, and radar hardware components in the CW radar, this study assumed that the time period of the polynomial fitting in the proposed detrending technique was normally 60 s and less than 120 s at the maximum. The polynomial order in the proposed technique was adjusted from the second to the ninth order to determine the optimum order in a given time period.

### 2.3. Signal Processing of the CW Radar, including the Proposed Technique

**Figure 4:**
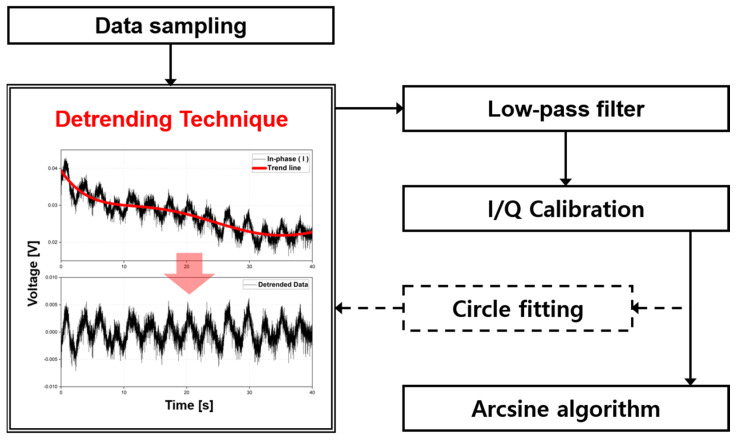
Overall flow chart for the digital signal processing, including the proposed detrending technique for the phase extraction of the CW radar.

The phase difference between the transmitting (Tx) and receiving (Rx) signals is obtained from the baseband signals in the I/Q channels of the CW radar using digital signal processing, including the proposed detrending technique. It is theoretically necessary for accurate phase extraction to simultaneously sample the I/Q baseband signals, and synchronous ADC is generally used in the data acquisition (DAQ) to neglect the phase variation between signal channels due to the switching time in the input channel of the ADC. When the phase variation in the baseband signals is sufficiently slow compared to the sampling rate of the ADC, a multichannel ADC with switching operations in the input channels can be used because the switching time delay between the input channels is not significant. The sampled signals were low-pass filtered using a cut-off frequency of approximately 5 Hz in the processing for vital sign detection and small displacement measurements. The cut-off frequency of the filter can be determined based on the frequency of the desired signals in the CW radar. The model equation for the DC drift was extracted by polynomial fitting with the second or higher order using the data obtained for less than 60 s. DC drift was removed by subtracting the value obtained from the model equation obtained from each sampled raw signal value. After processing with the proposed detrending technique, the amplitude and phase mismatches of I/Q signals due to hardware imperfections were calibrated by using the Gram–Schmidt procedure, which produces two orthonormal vectors as follows:

(6) $$\left[\begin{array}{c} I_{ort}\left(t\right)\\ Q_{ort}\left(t\right) \end{array}\right]=\left[\begin{array}{cc} 1 & 0\\ -\tan\varphi_{E} & \frac{A_{I}}{A_{Q}\cos\varphi_{E}} \end{array}\right]\left[\begin{array}{c} I\left(t\right)\\ Q\left(t\right) \end{array}\right]$$

where *I_ort_*(*t*) and *Q_ort_*(*t*) denote the calibrated baseband signals [31]. The calibrated I/Q baseband signals are displayed on the complex plane as a circle or an arc, depending on the length of the movement of the target object compared to the wavelength of the radar operating frequency. Circle fitting based on the Levenberg–Marquardt (LM) method can be used to check whether the proposed technique is effective in removing DC drift [18,32]. The correlation between the center of the generated circle and the origin of the complex plane showed that the DC drift was sufficiently removed by the proposed detrending technique. The phase information contained in the I/Q baseband signals is obtained using arcsine demodulation, which is a nonlinear demodulation method [33]. Figure 4 summarizes the overall flowchart of the proposed signal processing method for phase extraction in the CW radar.

## 3. Simulation Results

**Figure 5:**
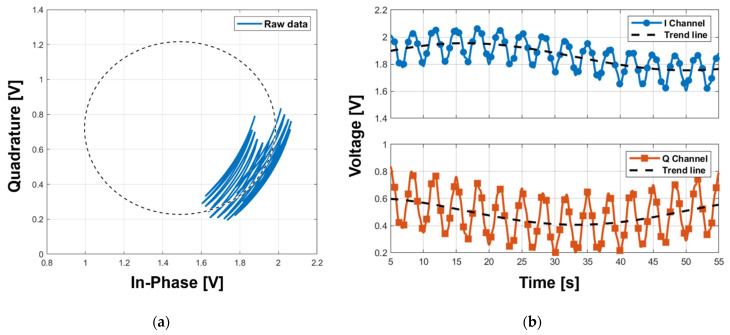
Baseband I/Q signals generated in the simulation to verify the proposed detrending technique: (**a**) I/Q plots; (**b**) time-domain waveforms.

The proposed detrending technique was verified by simulation using MATLAB. The I/Q baseband signals with different amplitudes were generated by using (1) and (2) modeled with the vital signs, which represent a respiration signal at a frequency of 0.3 Hz and a heartbeat signal at a frequency of 1.3 Hz. In the model function using (1) and (2), the distance *d_0_* and the phase error φ_E_ were assumed to be 0.5 m and 0°, respectively. Without distinguishing the origin of the DC drift, the DC drift in the simulation was assumed to be a trigonometric function with a frequency of 0.03 Hz using (3) and (4) to describe the change in the DC offset at a very low frequency, and the static DC offset in each channel was determined to an arbitrary value within the dynamic range of the ADC. Each baseband signal was generated for 60 s, and a data set for each channel was constructed with a sampling rate of 1000 samples per second. Figure 5 shows the I/Q baseband signals generated from the simulation displayed in the complex plane and the time domain, respectively. The baseband signals with the displacement, which have shorter wavelengths than those of the 5.8 GHz radar frequency, are displayed as arcs on the I/Q plot due to DC drift as shown in Figure 5a and as time-variant signals modulated with a very low frequency in the time domain as shown in Figure 5b.

**Figure 6:**
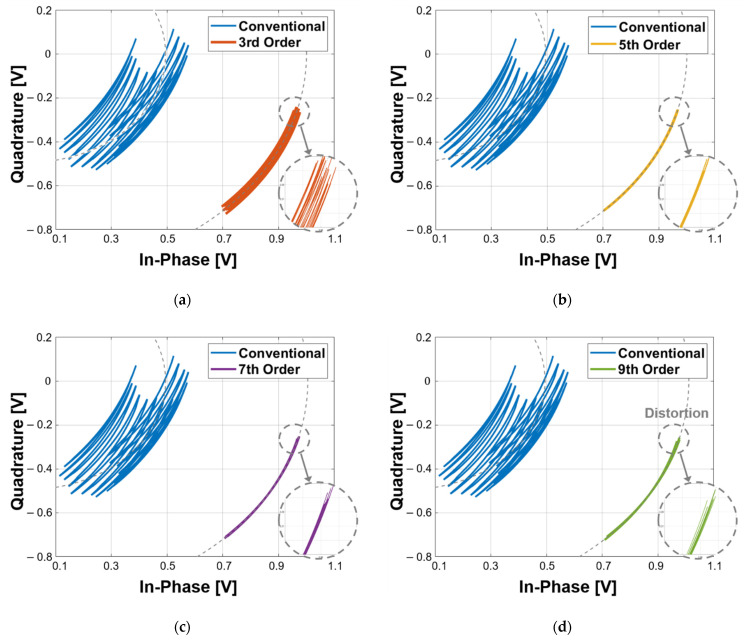
Simulation results using the conventional method for cancelling static DC offset and the proposed detrending technique using: (**a**) 3rd-order polynomials; (**b**) 5th-order polynomials; (**c**) 7th-order polynomials; (**d**) 9th-order polynomials.

Figure 6 shows the simulation results of the comparison between the conventional DC offset cancellation and the proposed detrending technique. The data obtained using the conventional method, which only removes a static DC offset, did not converge to the trajectory of a single circle, and the center of each circle was not the same as the origin of the complex plane. However, the data obtained using the proposed technique converged to a single trajectory of a single circle centered at the origin. A comparison of the characteristics of the proposed technique depending on the fitting order shows that the fitting data using the fifth- and seventh-order polynomials were located more accurately on the trajectory of the single circle than the data using the third- and ninth-order polynomials. The arcs obtained using the proposed technique with the third-order polynomial fitting are distributed near the trajectory of the single circle, as shown in Figure 6a, showing that the DC drift was not sufficiently removed compared to that in the technique with the other orders. This means that the DC drift for 60 s generated in the simulation cannot be sufficiently eliminated by the modeled function with the third-order polynomial. Both ends of the trajectory obtained using the ninth-order polynomial were wider than the midpoint of the trajectory, as shown in Figure 6d, which can be attributed to over-fitting distortion. The trajectories generated using the fifth- and seventh-order polynomials in Figure 6b,c have high accuracy and similarity. The simulation results, which depend on the order of the polynomial fitting in Figure 6, show that the proposed detrending technique has an optimum order for DC drift cancelation. However, the optimum order of the polynomial fitting can be changed by the DC drift waveforms, the data acquisition time (*T_DAQ_*), and the sampling rate due to the dependency of the best fitting polynomial.

**Figure 7:**
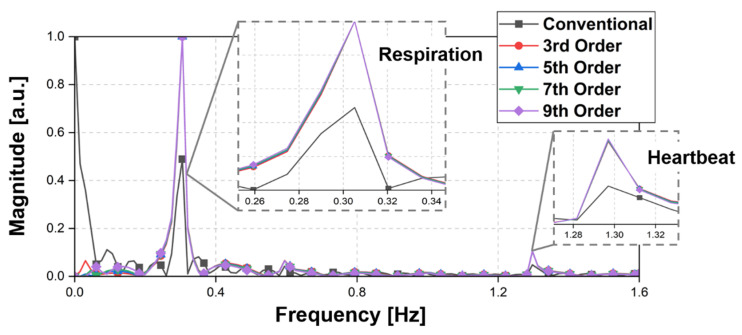
Simulation spectrum processed from the modeled waveforms by using the conventional method and the proposed detrending technique.

The simulation results in the frequency domain, which were obtained using the fast Fourier transform (FFT) as shown in Figure 7, show that the SNR of the vital signal detection modeled in the simulation can be improved by the proposed detrending technique. The proposed detrending technique increased the normalized magnitude of the desired signal compared to the conventional method, because the noise related to DC drift is significantly reduced by the proposed technique. The SNRs of the respiration and heartbeat signals were increased by 6.2 dB and 6.9 dB, respectively. The SNR improvement did not show a significant difference in the proposed technique when third-order or higher polynomial fitting was used. The simulation results showing the SNR improvement regardless of the order of the polynomial fitting demonstrate that the proposed technique using polynomials of a certain order or higher is sufficiently useful in the application without finding the best fitting order.

## 4. Experiment Setup

### 4.1. CW Radar Sensor Module

**Figure 8:**
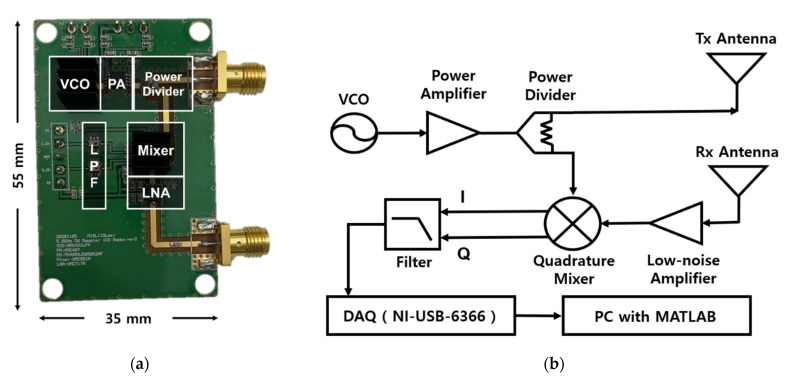
The 5.8 GHz CW radar sensor module: (**a**) photograph of the module implemented on an FR4 PCB; (**b**) block diagram of the module, including antennas, a DAQ board, and a PC.

The radar front-end circuit was implemented on an FR4 printed circuit board (PCB) with a thickness of 0.6 mm as shown in Figure 8a. Figure 8b shows the block diagram of the CW radar sensor operating at the 5.8 GHz ISM band. A 5.8 GHz signal was generated by a voltage-controlled oscillator (VCO) (HMC431LP4, Analog Devices Inc.) with an output power of 2 dBm and amplified by a power amplifier (HMV407MS8G, Analog Devices Inc.) with a power gain of 15 dB. A power divider (PD4859J5050S2HF, Anaren Inc.) was used to distribute the transmitted and local oscillator (LO) signals. The output power at the transmitter port was measured to be 10 dBm due to path loss. The receiver path consisted of a low-noise amplifier (HMC717ALP3E, Analog Devices Inc.), a down-conversion mixer (HMC951A, Analog Device Inc.), and low-pass filters (0500LP15A500, Johanson Technology). The total noise figure and gain of the receiver path were calculated to be 1.37 dB and 27.5 dB, respectively. The antenna gain of the patch antenna designed on an FR4 PCB with a thickness of 1 mm was measured to be 4.4 dBi [34]. The module size was set to 35 mm × 55 mm, excluding the Tx/Rx patch antennas. 

### 4.2. Data Acquisition from the Radar and Reference Sensors

The baseband I/Q signals were captured by a synchronous multichannel DAQ board (NI USB-6366, National Instruments) with sampling rates of 1k samples per second in each channel. The sampling rate was fixed in the experiment based on the simulation results of the proposed method, and the *T_DAQ_* of the proposed detrending method was initially set to approximately 60 s considering the characteristics of the target object. Digital signal processing, including the proposed detrending technique, was implemented using MATLAB on a PC. The performance of the CW radar using the conventional method and the proposed technique was demonstrated by a displacement measurement using a linearly moving stage and vital signal (respiration and heartbeat signals) detection for the subject. 

**Figure 9:**
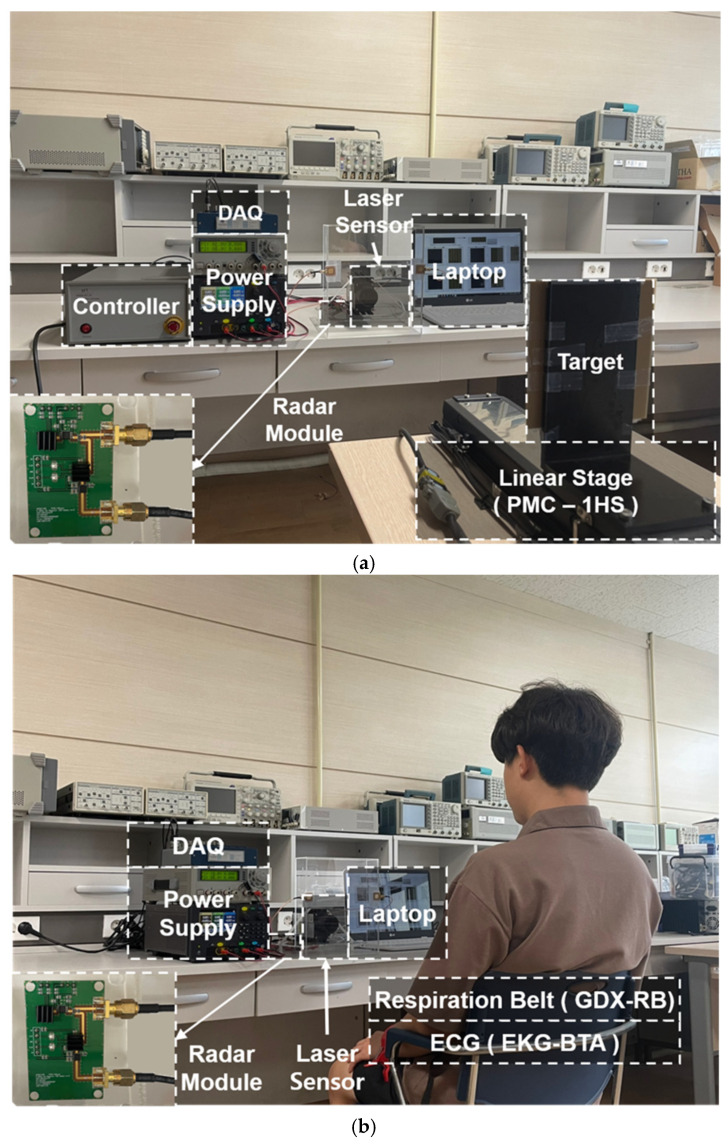
Experiment setup using the radar sensor: (**a**) displacement measurement; (**b**) vital signal detection.

In the displacement measurement, the target object periodically traveled within the range of 26 mm, which is half of the wavelength of 5.8 GHz, at a distance of 1 m by the moving stage with a range resolution of 1 µm as shown in Figure 9a. The target object and the antennas were placed on the same horizontal plane at a height of 1 m from the floor in order for the radar to measure the same displacement controlled by the moving stage. The range finder (ILR-1182, Micro-Epsilon) based on a laser with a resolution of 0.1 mm was used as the reference for the displacement measurement. The measured data in the radar were synchronized after capturing in each DAQ board due to the different sampling rates between the radar and reference sensors. The *T_DAQ_* for processing the proposed technique in the displacement measurement was modified to 120 s, which is longer than that in the simulation, because it was expected that the DC drift can be effectively removed by the proposed detrending as the time increased. It is difficult to determine the performance degradation in the DC drift caused by unwanted movement in the displacement measurement using the radar due to the precise displacement control of the moving stage. Therefore, the displacement was set to a half-wavelength of the operating frequency for the 5.8 GHz radar to draw a circle in the I/Q plot, and the long *T_DAQ_* was set such that the DC drift in the radar module itself could be generated due to heat or vibration.

The respiration and heartbeat signals were measured for the subject trying to maintain a still state by using the radar module at a distance of 0.8 m. Two contact sensors were used to determine the measurement accuracy of the vital signal detection: the respiration belt GDX-RB manufactured by Vernier Software & Technology, which sampled the respiration data with a sampling rate of 20 Hz, and the three-electrode ECG sensor EKG-BTA manufactured by Vernier Software & Technology, which monitored the heart rate with a sampling rate of 200 Hz. The detection accuracy was compared by synchronizing the data post-processing using the same method as in the displacement measurement because the sampling rates of both the CW radar and the reference sensors are different. Despite the subject’s effort to maintain the posture for the measurement time, the generation of microscopic movements of the subject, such as moving the body back and forth or wiggling, cannot be avoided, and these movements can generate a DC drift with a very low frequency in the CW radar [28]. The proposed technique should be verified using a shorter *T_DAQ_* in the vital signal detection than the *T_DAQ_* in the displacement measurement because the DC drift generated by the unintentional movement of the human body is unpredictable and uncontrollable. Therefore, the *T_DAQ_* in the vital signal detection was arbitrarily set to 40 s, which is shorter than the 60 s verified in the simulation results.

## 5. Measurement Results and Discussions

### 5.1. Displacement Measurement

**Figure 10:**
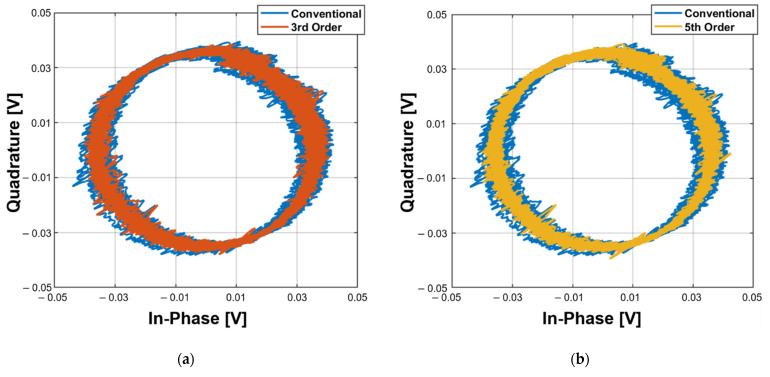

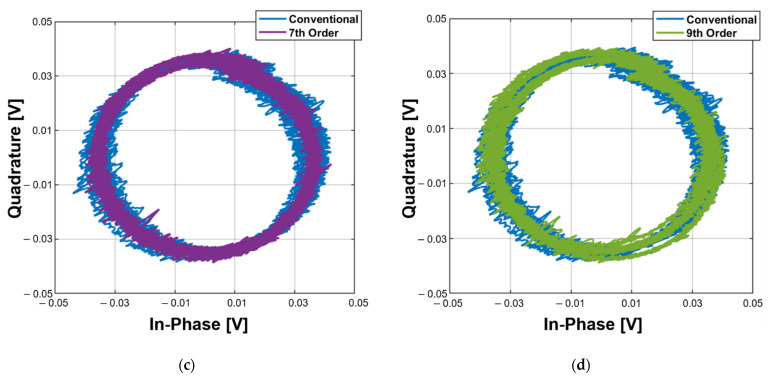
I/Q plots in the displacement measurement obtained by the circle fitting method after processing the conventional static DC offset cancellation and the proposed detrending technique depending on the order of the polynomial fitting: using the polynomial with (**a**) third order; (**b**) fifth order; (**c**) seventh order; (**d**) ninth order.

The accuracy of the displacement measurement shows that the proposed detrending technique can more effectively remove DC drift compared to the conventional method because the DC drift reduces the accuracy of detecting the phase difference between the Tx and Rx signals in the CW radar. The baseband signals obtained from the ideal single target with a half-wavelength displacement theoretically draw an I/Q plot as a single radius circle centered on the origin of the complex plane. The I/Q plot displayed with the measurement data might form several circles with different radii or deviated center points. To compare the performance of the conventional DC offset cancelation and the proposed detrending technique, the centers of the generated circles were fixed at the origin, and the accuracies of the displacement measurement using both methods were analyzed with the standard deviation (SD) of the radius. The I/Q plots to compare the proposed technique with the conventional method in Figure 10 show that the proposed technique can provide a more accurate single circle fitting by reducing the effect of the DC drift regardless of the fitting order.

**Figure 11:**
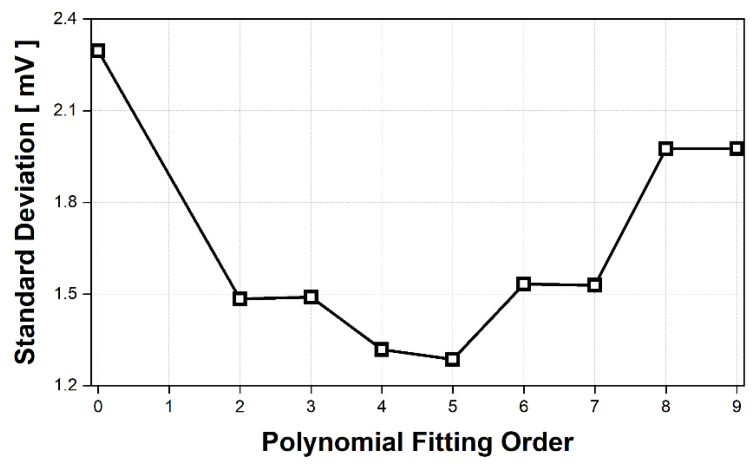
Standard deviation for the radius of the generated circle trajectory in the I/Q plots shown in Figure 10.

It can be regarded that the signal processing method with the smallest SD of the circle, that is, the technique with the highest accuracy of the circle fitting, offers the best performance in removing DC drift because the target in the measurement moves repeatedly in the range for the *T_DAQ_*. Figure 11 shows the standard deviation of the circle trajectory generated by the circle fitting method after processing the measurement data with the conventional static DC offset cancelation (no fitting, equivalent to order 0 in Figure 11) and the proposed technique (polynomial fitting with second to ninth order). Compared with the conventional method, the low SD of the proposed technique indicates that it is useful for improving the accuracy of phase detection by generating a highly accurate circle trajectory. In addition, Figure 11 shows that one polynomial fitting of the proposed technique was most effective in removing the DC drift. The best fitting displacement measurement was achieved using the fifth-order polynomial, which is a similar order to that presented in the simulation. Although the simulation and measurement results are based on data captured at different time durations, the similar orders of the polynomial fitting show that the proposed detrending technique using fifth-order polynomial fitting can be generally used to remove DC drift in simulation conditions and the experimental setup of CW radars.

**Figure 12:**
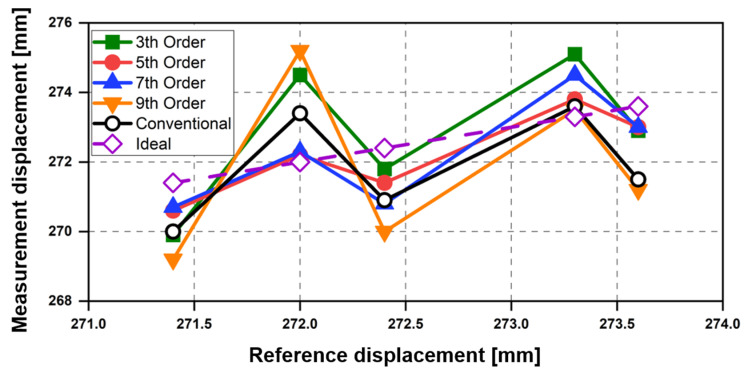
Measurement error in the displacement obtained by the CW radar depending on the DC offset cancellation method.

The measurement error in the displacement obtained by the radar, depending on the DC offset cancelation, is shown in Figure 12. The proposed technique using the fifth-order polynomial fitting, which shows the smallest SD in Figure 11, had the lowest error of 0.62 mm on average. These results show that the fifth-order polynomial was the best fit in the proposed technique. The average error in the displacement measurement was 1.34 mm when the conventional method was used. This was higher than the error obtained in the proposed technique when the fifth- and seventh-order (0.88 mm) polynomials were used but lower than those obtained when the third- (1.42 mm) and ninth-order (2.08 mm) polynomials were used. The increase in error using the third- and ninth-order polynomials indicates that the performance variation of the proposed technique might increase depending on the displacement when the DC drift is estimated with too low or high order polynomials. Therefore, the measurement results of the displacement demonstrate that the optimum order setup is important in the polynomial fitting of the proposed technique, and the fifth-order polynomial is the most effective in removing the DC drift in the experimental setup described in Section 4.

### 5.2. Vital Signal Detection

**Figure 13:**
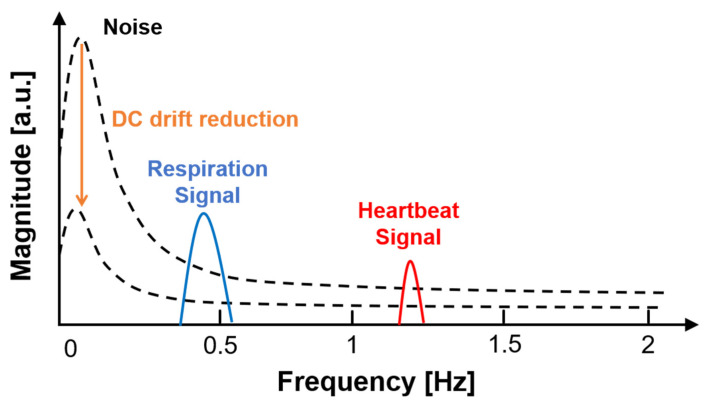
DC drift in the respiration and heartbeat detection using the CW radar.

The effect of DC drift on vital signal detection using the CW radar is shown in Figure 13. The DC drift in the baseband I/Q signals is represented as a low-frequency modulation signal in the time domain, which is caused by the complex combination of an undesired very slow motion from the radar or the target, temporal change in DC offset in the radar, and noise floor increase due to heat generation. When DC drift is reduced by the proposed technique, the overall noise characteristics in the baseband can be reduced, not just the low-frequency noise, resulting in an improved SNR of respiration and heartbeat signals as shown in Figure 13.

**Figure 14:**
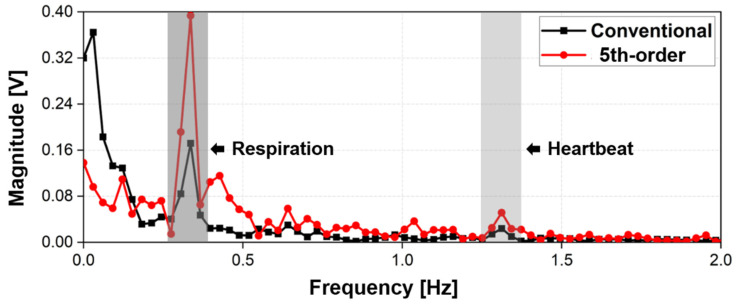
Measurement results of the vital signal detection using the 5.8 GHz CW radar processed by the conventional DC offset cancellation and the proposed detrending technique with the fifth-order polynomial fitting.

Figure 14 shows the frequency spectrum of the measured respiration and heartbeat signals using the 5.8 GHz radar with the conventional DC offset cancelation and the proposed technique. The proposed technique using only the fifth-order polynomial fitting was used for vital signal detection because the simulation and displacement measurement results showed that the fifth-order polynomial fitting was the best for removing DC drift in the proposed method. The measurement results in the frequency domain show that the proposed technique reduces the voltage magnitude due to DC offset and DC drift more than twice as much as the conventional method, and the decrease in the signals at DC and near DC coincides with the description in Figure 13. However, the magnitudes of respiration and heartbeat signals by the proposed technique increased in the measurement results compared to the conventional method, contrary to the description in Figure 13. The performance improvement of the proposed method, shown in Figure 14, can be explained by the improvement in the accuracy of the phase extraction in digital signal processing. The signal processing in Figure 4 shows that the phase extraction was performed after the DC offset and drift removal. The respiration and heartbeat signals in the raw data obtained from the baseband channels are independent of the method used to remove the DC offset and drift, but the accuracy of the phase extraction after I/Q calibration can vary depending on the degree of the DC offset and drift removal as mentioned in Section 2. 

The vital signals measured using the CW radar with the proposed technique had an accuracy of 98.89% in respiration and 99.54% in heartbeat as compared to the reference sensors. The accuracy difference depending on the polynomial order was not indicated in the experimental results because the same frequency peak of the vital signals was detected in the measurement regardless of the polynomial order in the proposed technique. The difference in the SNR between the conventional method and the proposed detrending technique can be expressed as the ratio of the magnitude of each vital signal shown in Figure 14, because the difference in the noise level due to DC offset outside the frequency band of interest is negligible. The SNR improvement of the vital signal detection was measured as 7.2 dB in respiration and 6.6 dB in heart rate, and the SNR of the respiration displayed in a lower frequency band was improved more than that of the heartbeat by the proposed technique. This is because the respiration signal was closer to the DC.

The simulation and measurement results show an improvement in the accuracy of the displacement measurement and the SNR in the vital signal detection by using the proposed technique in the CW radar. The results show that for a data acquisition time from 40 s to 120 s and a detection distance within 1 m, a polynomial fitting of the fifth order shows the best performance in the proposed technique. However, it is thought that the polynomial fitting order of the proposed technique can be sufficiently varied depending on the target object, measurement conditions, and surroundings because there is no theoretical basis that the fifth order would always be the best order of the polynomial fittings. It will be a good further research study for us to show whether the best order converges in more diverse targets and environments.

## 6. Conclusions

A detrending technique was proposed to remove the noise generated by the static and time-variant DC offset in a CW radar. DC drift, which is the time-variant DC offset, should be removed for accurate phase detection in CW radars. The proposed technique, which models DC drift using polynomial fitting, can remove DC drift in the time domain more effectively compared to the conventional method, which removes only the static DC offset or uses a high-pass filter. The simulation results for vital signal detection using the CW radar showed that the proposed technique effectively removes DC drift modeled with a frequency that is 10 times less than that of the respiration, and the effect of the DC drift removal can vary depending on the fitting order of the polynomial in the proposed technique. In the measurement using a 5.8 GHz CW radar, the accuracy of the displacement measurement and the SNR of the vital signal detection were improved by the proposed technique compared to the conventional method for removing the static DC offset. Compared to the conventional method, the proposed technique using the fifth-order polynomial fitting at a distance of 1 m or less showed an average improvement of 3.7 times in terms of the error in the displacement measurement, and the SNRs of the vital signal detections were increased by 7.2 dB and 6.6 dB for respiration and heartbeat, respectively. In addition, the simulation and measurement results showed that there is a best fitting order in the proposed technique, and the fifth-order fitting in the given simulation and measurement environment removed the effect of the DC drift most effectively.

## Data Availability

The data presented in this study are available on request from the corresponding author. The data are not publicly available due to the privacy of the subjects.

## References

[1] Iwata Y., Thanh H.T., Sun G., Ishibashi K. (2021). High accuracy heartbeat detection from CW-Doppler radar using singular value decomposition and matched filter. Sensors.

[2] Constanzo S. (2019). Software-defined Doppler radar sensor for human breathing detection. Sensors.

[3] Kim H., Jeong J. (2020). Non-contact measurement of human respiration and heartbeat using W-band Doppler radar sensor. Sensors.

[4] Kim J.-Y., Park J.-H., Jang S.-Y., Yang J.-R. (2019). Peak detection algorithm for vital sign detection using Doppler radar sensors. Sensors.

[5] Park J., Jeong Y., Lee G., Oh J., Yang J. (2019). 915-MHz Continuous-wave Doppler radar sensor for detection of vital signs. Electronics.

[6] Hyun E., Jin Y.-S., Park J.-H., Yang J.-R. (2020). Machine learning-based human recognition scheme using a Doppler radar sensor for in-vehicle applications. Sensors.

[7] Li C., Lubecke V., Boric-Lubecke O., Lin J. (2013). A Review on recent advances in Doppler radar sensors for noncontact healthcare monitoring. IEEE Trans. Microw. Theory Tech..

[8] Gennarelli G., Ludeno G., Soldovieri F. (2016). Real-time through-wall situation awareness using a microwave Doppler radar sensor. Remote Sens..

[9] Hong H., Zhang L., Gu C., Li Y., Zhou G., Zhu X. (2018). Noncontact sleep stage estimation using a CW Doppler radar. IEEE J. Emerg. Sel. Top. Circuits Syst..

[10] Sim J.Y., Park J.-H., Yang J.-R. (2020). Vital-signs detector based on frequency-shift keying radar. Sensors.

[11] Wang J., Karp T., Muňoz-Ferreras J.-M., Gómez-García R., Li C. (2019). A spectrum-efficient FSK radar technology for range tracking of both moving and stationary human subjects. IEEE Trans. Microw. Theory Tech..

[12] Park J.-H., Yang J.-R. (2020). Two-tone continuous-wave Doppler radar based on envelope detection method. Microw. Opt. Technol. Lett..

[13] Kuutti J., Paukkunen M., Aalto M., Eskelinen P., Sepponen R.E. (2015). Evaluation of a Doppler radar sensor system for vital signs detection and activity monitoring in a radio-frequency shielded room. Measurement.

[14] Yang J.-R., Kim D.-W., Hong S. (2007). A calibration method of a range finder with a six-port network. IEEE Microw. Wirel. Compon. Lett..

[15] Singh A., Gao X., Yavari E., Zakrzewski M., Cao X., Lubecke V., Boric-Lubecke O. (2013). Data-based quadrature imbalance compensation for a CW Doppler radar system. IEEE Trans. Microw. Theory Tech..

[16] Kim D.K., Kim Y. (2019). Quadrature frequency-group radar and its center estimation algorithms for small vibration displacement. Sci. Rep..

[17] Park J.-H., Yang J.-R. Multiphase continuous-wave Doppler radar with multiarc circle fitting algorithm for small periodic displacement measurement. IEEE Trans. Microw. Theory Tech..

[18] Zakrzewski M., Raittinen H., Vanhala J. (2012). Comparison of center estimation algorithms for heart and respiration monitoring with microwave Doppler radar. IEEE Sens. J..

[19] Gao X., Boric-Lubecke O. (2017). Radius correction technique for Doppler radar noncontact periodic displacement measurement. IEEE Trans. Microw. Theory Tech..

[20] Gao X., Singh A., Yavari E., Lubecke V., Boric-Lubecke O. Non-contact displacement estimation using Doppler radar. Proceedings of the Annual International Conference of the IEEE Engineering in Medicine and Biology Society.

[21] Park B., Boric-Lubecke O., Lubecke V. (2007). Arctangent demodulation with DC offset compensation in quadrature Doppler radar receiver systems. IEEE Trans. Microw. Theory Tech..

[22] Vergara A., Boric-Lubecke O., Lubecke V. DC information preservation for cardiopulmonary monitor utilizing CW Doppler radar. Proceedings of the Annual International Conference of the IEEE Engineering in Medicine and Biology Society.

[23] Li S., Xiong Y., Ren Z., Gu C., Peng Z. Ultra-micro vibration measurement method using CW Doppler radar. Proceedings of the International Conference on Sensing, Measurement & Data Analytics in the era of Artificial Intelligence (ICSMD).

[24] Svitek R., Raman S. (2005). DC offsets in direct-conversion receivers: Characterization and implications. IEEE Microw. Mag..

[25] Kim Y., Kim D. Experimental characterization process of DC-offset performance for multiple CW Doppler radar. Proceedings of the International Technical Conference on Circuits/Systems, Computers and Communications (ITC-CSCC).

[26] Kuo H., Chou C., Lin C., Yu C., Huang T., Chuang H. A 60-Ghz CMOS direct-conversion doppler radar RF sensor with clutter canceller for single-antenna noncontact human vital-signs detection. Proceedings of the 2015 IEEE Radio Frequency Integrated Circuits Symposium (RFIC).

[27] Pan J., Wang Z., Tang S., Wang J., Li C., Huangfu J., Ran L. DC offset and low frequency noise compensation for direct-conversion receiver in pulse compression radar. Proceedings of the International Workshop on Antenna Technology (iWAT).

[28] Lv Q., Chen L., An K., Wang J., Li H., Ye D., Huangfu J., Li C., Ran L. (2018). Doppler vital signs detection in the presence of large-scale random body movements. IEEE Trans. Microw. Theory Tech..

[29] Kim Y., Kim D. Control the value of I/Q DC-bias and gain from the experimental result of 24GHz Doppler radar. Proceedings of the International Conference on Information and Communication Technology Convergence (ICTC).

[30] Park B., Lubecke V., Boric-Lubecke O., Host-Madsen A. Center tracking quadrature demodulation for a Doppler radar motion detector. Proceedings of the IEEE/MTT-S International Microwave Symposium.

[31] Park B., Yamada S., Lubecke V. (2007). Measurement method for imbalance factors in direct-conversion quadrature radar systems. IEEE Microw. Wirel. Compon. Lett..

[32] Schweizer B., Knill C., Schindler D., Waldschmidt C. IQ-imbalance compensation for wideband OFDM-radar. Proceedings of the European Conference on Antennas and Propagation (EuCAP).

[33] Fan T., Ma C., Gu Z., Lv Q., Chen J., Ye D., Huangfu J., Sun Y., Li C., Ran L. (2016). Wireless hand gesture recognition based on continuous-wave Doppler radar sensors. IEEE Trans. Microw. Theory Tech..

[34] Jeong J.-I., Yang J.-R. 5.8-GHz patch antenna with enhanced defected ground structure for size reduction and increased bandwidth. J. Electromagn. Eng. Sci..

